# Diagnosis of Dengue Virus Infections Imported to Hungary and Phylogenetic Analysis of Virus Isolates

**DOI:** 10.3390/diagnostics13050873

**Published:** 2023-02-24

**Authors:** Orsolya Nagy, Anna Nagy, Anita Koroknai, Nikolett Csonka, Mária Takács

**Affiliations:** 1 National Reference Laboratory for Viral Zoonoses, National Public Health Center, 1097 Budapest, Hungary; 2 Institute of Medical Microbiology, Semmelweis University, 1085 Budapest, Hungary

**Keywords:** Dengue virus, virus isolation, whole-genome sequencing, imported arbovirus infection

## Abstract

Background: Dengue virus is one of the most important arbovirus infections of public health concern. Between 2017 and June 2022, 75 imported dengue infections were confirmed by laboratory diagnostic methods in Hungary. Our study aimed to isolate the imported Dengue strains and characterize them by whole-genome sequencing. Methods: Laboratory diagnosis of imported infections was carried out using both serological and molecular methods. Virus isolation was attempted on Vero E6 cell lines. An in-house amplicon-based whole-genome sequencing method was applied for the detailed molecular characterization of the isolated virus strains. Results: From 75 confirmed Dengue infected patients, 68 samples were used for virus isolation. Isolation and whole-genome sequencing were successful in the case of eleven specimens. Isolated strains belonged to Dengue-1,-2,-3 serotypes. Discussion: The isolated strains corresponded to the circulating genotypes of the visited geographic area, and some of the genotypes were linked with more severe DENV cases in the literature. We found that multiple factors, including viral load, specimen type, and patient antibody status, influence the isolation efficacy. Conclusions: Analysis of imported DENV strains can help estimate the outcomes of a possible local DENV transmission in Hungary, a threat from the near future.

## 1. Introduction

Dengue virus (DENV) is a member of the Flaviviridae family, Flavivirus genus, and has four serotypes (DENV-1, DENV-2, DENV-3, and DENV-4) that are antigenically closely related. A fifth serotype (DENV-5) was discovered in Malaysia in 2007, but its importance remains unclear [1]. DENV is one of the most important arbovirus infections of public health concern in tropical and subtropical regions and is responsible for an estimated 390 million infections yearly [2]. Any of the four serotypes of dengue can cause a wide spectrum of disease: most infections remain asymptomatic or have mild manifestation, also known as dengue fever (DF), with fever, headache, retro-orbital pain, myalgia, arthralgia, and rash symptoms. Elevated transaminases, leucopenia, and mild thrombocytopenia may also be observed in DF. Rarely, dengue hemorrhagic fever (DHF), a more severe form of the disease, develops, characterized by coagulopathy, increased vascular permeability, and mucosal bleeding. It can progress into hypovolemic shock of dengue shock syndrome (DSS) [2,3,4]. Antibody-dependent enhancement of infectivity (ADE), a phenomenon typical to Flaviviruses, is frequently observed in the background of severe dengue. In this case, pre-existing cross-reactive but non-neutralizing or poorly neutralizing antibodies from a previous infection caused by a different DENV serotype, or an antigenically closely related flavivirus, lead to enhanced infection [5,6,7]. Dengue is transmitted by female *Aedes* mosquitoes, mainly *Aedes aegypti*, but *Aedes albopictus* can also be involved in transmission between humans [8]. Since these mosquitoes occur in certain regions of Europe [9], an imported infection poses a risk for autochthonous spreading in the months of vector activity [10]. No specific antiviral treatment is available for Dengue. Case management is based on symptomatic therapy, and patients with severe DENV infections need hospitalization for adequate supportive care [2,3,4]. The implementation of personal protective measures such as using insect repellents, mosquito nets, and wearing long-sleeved clothing to avoid mosquito bites is recommended for travelers of DENV endemic regions [2,3,4]. Dengvaxia, a chimeric yellow fever-dengue-tetravalent dengue vaccine (CYD-TDV), is licensed in the EU for people aged between 6 and 45 years who have had the infection in the past. However, people who are immunologically naïve to dengue infection may have a higher risk of severe dengue disease if they become infected with the virus following vaccination with Dengvaxia. Therefore, the vaccine should only be administered to people who have had a previous, test-confirmed dengue infection [11,12].

The laboratory diagnosis of acute DENV infections is based on both serological and molecular techniques. The demonstration of specific antibodies from paired sera and the detection of NS1 antigen from blood collected 0 to 9 days after symptom onset are among the most widely used diagnostic methods [13]. Viral RNA is detectable in serum or anticoagulated whole blood collected during the very early phase of the infection, and may be traceable longer in urine [14,15]. Virus isolation can be attempted from acute-phase clinical specimens. Laboratory diagnosis is, overall, challenging because viremia is short, and antibodies cross-react with other flaviviruses [13,16,17]. For this reason, during the serological diagnosis of DENV infections, a possible co-infection with other circulating flaviviruses or previous flavivirus infections and/or vaccination should be taken into account.

Although Hungary is found in the climatically temperate zone of Europe, *Aedes albopictus*, one of the competent vectors of DENV, is established in the southern Zala and Somogy counties and the central Pest County [9]. Since the introduction of serological methods during the first years of the 21st century, and the introduction of DENV-specific real-time RT-PCR in 2015, only imported cases were reported in the country. Prior to our study, no phylogenetic analysis of imported DENV strains from clinical specimens or isolates was described in Hungary.

Our study was conducted to characterize DENV strains from virus isolates of imported DENV infections between 2017 and June 2022. Two flaviviruses, tick-borne encephalitis virus (TBEV) and West Nile virus (WNV), are endemic in Hungary and cause human infections annually [18]. Human Usutu virus (USUV) infection was also diagnosed for the first time in 2018 [19]. Although no autochthon vector-transmitted DENV infections were reported from Hungary, with the presence of competent vectors the epidemiological situation may change in the near future. We believe that the detailed characterization of imported DENV strains by whole-genome sequencing and phylogenic analysis can help to understand the outcomes of a possible local circulation.

## 2. Materials and Methods

Laboratory diagnosis of DENV infections in Hungary is centralized and carried out exclusively at the National Reference Laboratory for Viral Zoonoses at the National Public Health Center, according to the decree of the Ministry of Health 18/1998 (XII 27). Between January 2017 and June 2022, 758 patients altogether with recent travel history to DENV endemic regions and clinical symptoms characteristic of DENV were treated at different hospitals and out-patient centers of the country. More than 90% of patients resided in the capital or in neighboring areas in Pest County, where *Aedes albopictus*, one of the competent vectors of DENV, is established [9]. For laboratory diagnosis, different specimen types—serum, and, when available, whole blood and urine—were collected from the patients. Samples were tested with serological and/or molecular techniques at the National Reference Laboratory. Alongside DENV, parallel testing for Zika virus (ZIKV) and Chikungunya virus (CHIKV) was also carried out because of the shared endemic regions and similar clinical presentation of the three viruses. In the case of reactive antibody results, serological tests for endemic flaviviruses of Hungary were also performed to exclude cross-reactivity. Patients’ flavivirus vaccination status was also taken into account when investigating cross-reactivity.

The detection of DENV, ZIKV and CHIKV specific antibodies was carried out from sera by immunofluorescence assay (Arbovirus Fever Mosaic 2 from Euroimmun Medizinshe Labordiagnostika, Lübeck, Germany) and/or ELISA (Euroimmun Medizinshe Labordiagnostika, Lübeck, Germany). DENV NS1 antigen was tested from sera collected 0 to 9 days past symptom onset with ELISA (Euroimmun Medizinshe Labordiagnostika, Lübeck, Germany). An in-house and/or commercially available immunofluorescence assay (Flavivirus Mosaic from Euroimmun Medizinshe Labordiagnostika, Lübeck, Germany) was used to exclude cross-reactivity caused by endemic flaviviruses of Hungary or previous flavivirus vaccination. Viral RNA detection was attempted from sera, and when available from anticoagulated whole-blood and urine specimens. Total nucleic acid was extracted from a 140 µL specimen using QIAamp Viral RNA Mini Kit (QIAGEN, Hilden, Germany). Amplification by real-time reverse-transcription (RT) PCR was performed following the previously reported method [20]. DENV serotype identification was also carried out using this technique.

The case evaluation of patients was carried out following the laboratory criteria described in EU case definitions, as published in the Official Journal of the European Union (Commission Implementing Decision (EU) 2018/945) [21]:

Probable case:

− Detection of dengue-specific IgM antibodies in a single serum sample.

Confirmed case:

At least one of the following five:

− Isolation of a dengue virus from a clinical specimen;
− Detection of dengue viral nucleic acid from a clinical specimen;
− Detection of dengue viral antigen from a clinical specimen;
− Detection of dengue-specific IgM antibodies in a single serum sample AND confirmation by neutralization;
− Seroconversion or four-fold antibody titer increase of dengue-specific antibodies in paired serum samples.

Virus isolation from PCR-positive sera, whole-blood, and urine samples was performed in biosafety level 3 (BSL3) containment at the National Public Health Center of Hungary, on Vero E6 (ATCC) cell lines. Cells were grown in T25 tissue culture flasks and seeded in cell culture supports in Dulbecco modified Eagle medium (DMEM) (Gibco™) with 10% fetal bovine serum (FBS) (Gibco™) and Cell Culture Guard (1:100 dilution) (ITW Reagents, Milano, Italy). After 48 h, when the cells were at 70% to 90% confluence, a final volume of 0.5 mL/flask of sera or whole-blood diluted in 1 mL DMEM (Gibco™) and 2 mL of undiluted urine was inoculated onto the monolayers. Samples with insufficient volume or low viral load (Ct values higher than 35) were excluded from the study. Urine samples were filtered with 0.22 µm pore size membrane filters and treated with 0.01 M TRIS buffer to adjust the urine pH to 7.2 to 7.8 prior to inoculation. The flasks were incubated for 90 min at 37 °C in 5% CO_2_. After incubation, DMEM (Gibco™) with 10% FBS (Gibco™) and Cell Culture Guard (1:100 dilution) (ITW Reagents, Milano, Italy) was added to the cells, but the inoculum was not removed. Cells were then cultured at 37 °C in 5% CO_2_ for up to 7 days and the presence of cytopathic effect (CPE) was monitored daily. After 7 days, a blind passage was carried out and incubation continued for another 7 days, with monitoring of CPE. After day 14, cells were harvested and real-time RT-PCR was performed from the supernatant using the previously described method [20] to detect increased DENV RNA load. The commercially available RealStar^®^ DENV RT-PCR kit 2.0 (Altona Diagnostics GmbH, Hamburg, Germany) was also performed on isolated strains to further confirm the presence of DENV RNA.

Amplicon-based whole-genome sequencing (WGS) of DENV isolates was carried out to characterize genetic variations. For nucleic acid extraction, we used the QIAamp Viral RNA Mini Kit (QIAGEN, Hilden, Germany), and then a one-step RT-PCR was carried out using 5 overlapping genomic fragments (5 amplicon/specimens) to amplify the whole genome of DENV, with different primer sets for each DENV serotype, according to our in-house protocol [20]. For post-PCR size section clean-up, we used reagents of Agencourt AMPure XP (Beckman Coulter). DNA concentration was determined by Qubit™ 1x dsDNA High Sensitivity (HS) Kit (Invitrogen™) and Qubit Flex Fluorometer (Invitrogen™), followed by equimolar pooling of the PCR amplicons/specimens. DNA library preparation was performed by Nextera XT DNA Library Preparation Kit (Reference Guide 15031942v06) (Illumina, Inc., San Diego, CA, USA), following the manufacturer’s instructions. Sequencing was carried out on the Illumina MiSeq platform with MiSeq Reagent Micro kit, v2, 300 cycles (MS-103-1002) (Illumina, Inc., San Diego, CA, USA). For quality control of the fastq files Genome Detective Virus Tool version 2.41 was used [22], and for genotype determination the Dengue Virus Typing Tool of the Genome Detective was applied [23]. For reference mapping of the sequence reads by Bowtie 2 mapping algorithm, UNIPRO UGENE 38.1 software was used. To compare assembled genome sequence similarities to sequences uploaded to the NCBI GenBank (National Center for Biotechnology Information) [24], pairwise alignment was performed by BLAST (Basic Local Alignment Search Tool) [25].

For phylogenetic analysis, sequences were aligned by MUSCLE (MUltiple Sequence Comparison by Log-Expectation) [26]. Editing of aligned sequences was carried out by GeneDoc 2.7 software [27]. Phylogenetic maximum likelihood tree was created by MEGA11 (Molecular Evolutionary Genetics Analysis) software (version 11) [28]. One thousand replicates for bootstrap testing were generated. The evolutionary distance was calculated using the General Time Reversible Model.

## 3. Results

**Figure 1:**
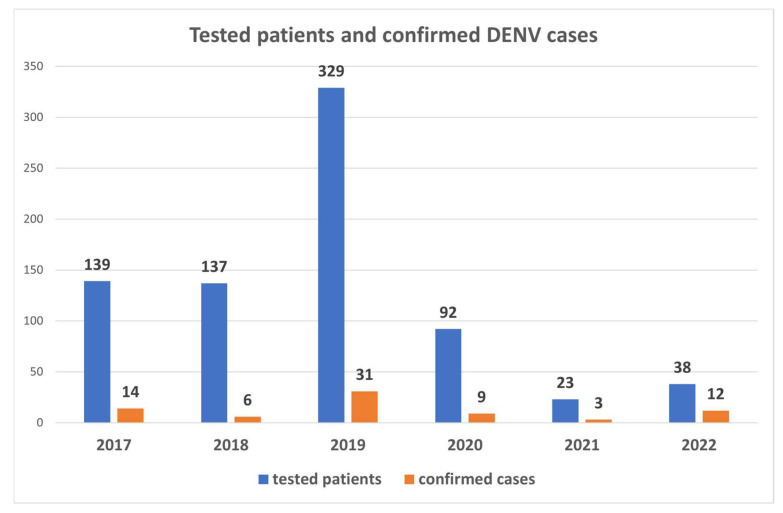
Tested patients and confirmed DENV cases/year between 2017 and June 2022.

**Figure 2:**
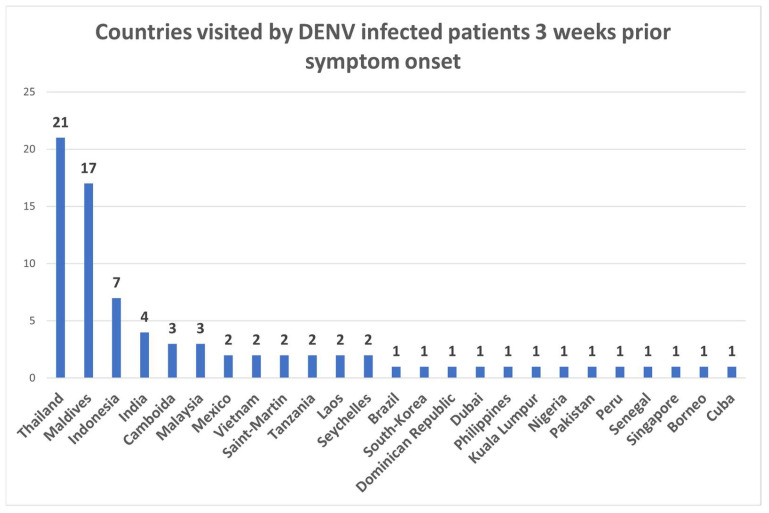
Countries visited by confirmed DENV-infected patients 3 weeks before symptom onset.

According to the decree of the Ministry of Health, 18/1998 (XII 27) DENV, ZIKV and CHIKV are notifiable diseases in Hungary; therefore, all symptomatic cases with a travel history to endemic regions must be tested by laboratory methods. Based on the results of the performed tests, DENV was the most frequently imported arbovirus infection to Hungary between 2017 and July 2022, followed by CHIKV (six confirmed cases) and ZIKV (three confirmed cases). Out of the tested 758 symptomatic patients, DENV infection was confirmed following the EU case definitions [21] in 75 cases by serological and/or molecular methods. A total of 41 (54.66%) patients were women, 34 (45.33%) were men, and the median age was 39 (range 15–65 years). The majority of the cases developed symptoms characteristic of DF, and three patients (4%) reported mucosal bleeding. The most frequent symptoms included fever (98.66%, 74 patients), rash (57.33%, 43 patients), myalgia (26.66%, 20 patients), headache (24%, 18 patients), and arthralgia (21.33%, 16 patients). Gastrointestinal (18.66%, 14 patients) and respiratory (9.33%, 7 patients) complications, thrombocytopenia (9.33%, 7 patients), leucopenia (6.66%, 5 patients) and elevated liver enzymes (6.66%, 5 patients) were also among the common manifestations. Based on laboratory results, six cases were evaluated as secondary DENV infections. Eight patients received vaccination against flaviviruses: four travelers were vaccinated against the yellow fever virus, and four against tick-borne encephalitis virus. No co-infections with other circulating arboviruses were identified. Most imported DENV infections were detected in 2019, while during the SARS-CoV-2 pandemic case numbers dropped significantly as a result of travel restrictions (Figure 1). Considering the travel history of DENV-infected patients in three weeks before symptom onset, most DENV cases were imported from 1. Thailand, 2. the Maldives and 3. Indonesia (Figure 2). Almost all imported cases were diagnosed between November and April, in accordance with the season of tropical vacations.

As different sample types were collected from the patients, viral RNA was detectable in 97 samples of the confirmed 75 cases: 51 sera, 29 whole-blood, and 17 urine samples. Based on sample volume and Ct values, out of the 97 specimens 68 (39 sera, 17 whole-blood, and 12 urine samples) were used for isolation. Virus isolation and whole-genome sequencing were successful in the case of 11 samples (nine sera and two whole-blood samples). All 11 specimens were collected 0–6 days post-symptom onset. The clinical presentation of most cases was characteristic of DF; one patient (9.1%) reported mucosal bleeding. Four isolates (36.36%) originated from male patients, and seven (63.63%) from female travelers, and the median age was 33 (range 15–63 years). Symptoms, travel history and serological results of the patients are summarized in Table 1. The majority of the isolated strains belonged to DENV-1 (54.54%, six isolates). DENV-2 (27.27%, three isolates) and DENV-3 (18.18%, two isolates) serotypes were also represented, while none of the isolated viruses belonged to DENV-4. Isolated genotypes corresponded >99% to the genotypes circulating at the visited geographical regions. Most isolates (27.27%, three isolates) were imported from the Maldives, from where the isolation of two different serotypes was successful: DENV-3 Genotype III was isolated in 2019, while DENV-2 Genotype II was isolated in 2022. Two samples with IDs 34/2021 and 48/2021 were collected from patients who traveled together and were infected at the same time in Saint-Martin; this explains the similarity of the isolated strains. No correlation between the DENV serotypes and genotypes with disease severity could be established due to the low number of available isolates, and because all patients except one presented with mild symptoms. One patient developed mucosal bleeding, but as he had received the TBEV vaccination previously the severity of the symptoms could also be explained by ADE.

**Figure 3:**
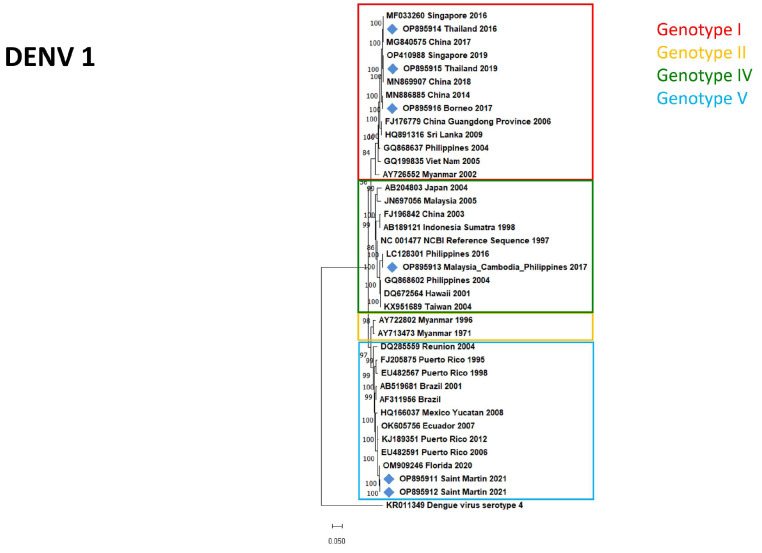
Maximum likelihood phylogenetic tree of DENV-1 strains. Isolates of imported DENV infections to Hungary marked by blue rhombuses.

**Figure 4:**
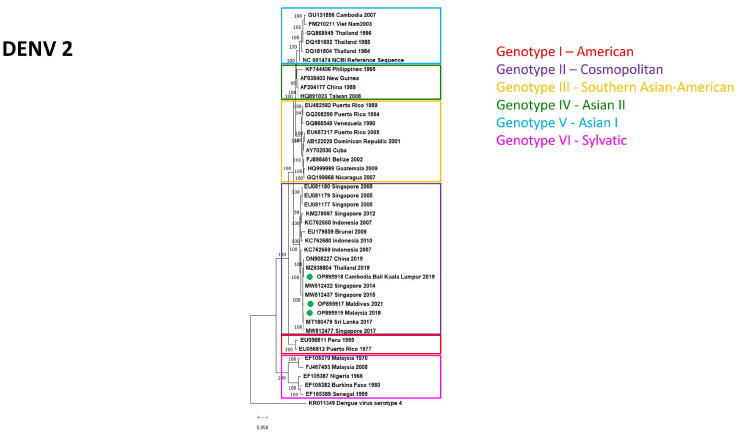
Maximum likelihood phylogenetic tree of DENV-2 strains. Isolates of imported DENV infections to Hungary marked by green circles.

**Figure 5:**
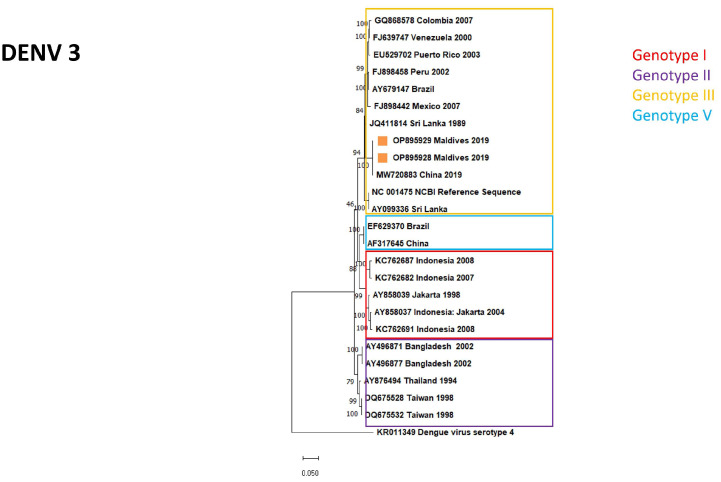
Maximum likelihood phylogenetic tree of DENV-3 strains. Isolates of imported DENV infections to Hungary marked by orange squares.

The evolutionary relationship between isolates and the circulating DENV genotypes is represented in Figure 3, Figure 4 and Figure 5 for serotypes DENV-1, DENV-2, and DENV-3, respectively. Information obtained by whole-genome sequencing is summarized in Table 2 and in the Supplementary Material.

## 4. Discussion

Previous studies tracking dengue epidemics highlight the role of viral evolution in replication fitness, pathogenicity, and infection dynamics [29,30,31,32,33,34,35]. Considering vaccine development and anti-viral treatment research, it is important to map and follow the circulating genotypes of endemic regions and to identify the genetic variations that help the positive selection of certain DENV strains. Until recently, no DENV isolates from clinical specimens of imported infections and no whole-genome sequencing of clinical isolates were reported in the literature from Hungary. In our work, we describe the first phylogenetic characterization of imported DENV genotypes of Hungarian patients. The identified strains were compared with the circulating genotypes of the visited geographical regions. Based on the sequencing data, isolated strains showed >99% similarity with the local genotypes. We also searched the literature for the dominant genotypes of the visited areas. According to reports on the phylogenetic analysis of circulating DENV strains in South Asia, from where most of our isolates were imported, all four DENV serotypes were co-circulating in recent years in the region, with the most dominant strains being Cosmopolitan genotype DENV-2, followed by genotype I DENV-1. The less dominant serotype was DENV-4, although serotype prevalence varied by country [36,37]. Based on a summary of imported DENV infections into the United States, the most frequently visited areas outside of the US were the Caribbean and Asia. Here, the majority of imported strains belonged to DENV-1, followed by DENV-2 and DENV-3; the less commonly imported serotype was DENV-4 [38]. In recent studies focusing on imported DENV infections to Italy, DENV-1 proved to be the most frequently imported serotype, followed by DENV-2, and the most visited areas were Asia and the Caribbean [39,40]. These findings are in accordance with our results of isolated DENV serotypes from Asia and the Caribbean, although to further map the actual prevalence of dominant genotypes more DENV strains need to be phylogenetically characterized.

Some of the isolated genotypes of Hungarian patients were previously linked to serious disease outcomes. Based on information found in the literature, the DENV-2 Cosmopolitan Genotype was associated with severe dengue outbreaks in Malaysia. Patients infected with this genotype were more commonly presented with plasma leakage and shock [41]. The DENV-3 Genotype III was also linked with higher rates of DHF in India [42]. The DENV-1 Genotype I has been described as causing large outbreaks with higher mortality rates and severe cases in many Asian countries, including India [43]. These findings predict that DENV cases with serious complications may also develop in the case of a potential local circulation. Moreover, due to the endemic flaviviruses in Hungary, co-infections and flavivirus antibodies from previous infections can also complicate the disease severity and lead to ADE. As laboratory diagnosis of DENV in Hungary is carried out only in case of symptomatic infections, our data are limited to the confirmed cases. Further genotypes associated with severe dengue might be identified with the testing of asymptomatic travelers. Alongside the circulating DENV strains of the visited geographical regions, many other factors of the visitors’ country, including vector dynamics, previous immunity, and socio-economic factors, determine the outcomes of possible local transmission, as has been modeled before [44].

When investigating the factors that might have influenced the efficacy of virus isolation, we found that viral load was relatively high in samples of successful isolation (Ct values ranging from 17.60 to 27.35). Out of the available urine specimens, 10 had significantly higher Ct values (above 30), which may refer to an insufficient viral load for isolation. As the shedding of DENV in the urine usually starts later and last longer than in serum or whole blood [15], and the time of sample collection happened 0 to 6 days after symptoms onset, this may indicate that the urine samples were collected at the beginning of the appearance of the virus particles. The viral load of serum and whole-blood specimens did not vary remarkably, but isolation was successful from notably more sera than from whole-blood samples, suggesting that serum is a more suitable specimen for virus isolation. This hypothesis needs further investigation. When comparing our findings with other studies, we found that the isolation of DENV from whole-blood specimens was successful using C6/36 mosquito cell lines, and at dilutions of 1:10 to 1:20 [14,45], much higher than the dilutions applied in our work. This may indicate that the C6/36 cell line is more permissive to DENV, and that in higher concentrations whole blood or the chemicals used for anticoagulation have a toxic effect on the cells that interferes with the procedure. Some studies found that whole blood is a more suitable specimen type, not only for RT-PCR but for virus isolation as well [45,46,47]. Other reports suggest that the detection of DENV RNA by RT-PCR is more sensitive if carried out from serum than from blood samples [48]. In addition, some studies concluded that virus isolation from whole blood is less effective than from serum or plasma [49]. These differences may originate from the diversity between the time of sample collection after symptom onset, and from the different isolation and RT-PCR protocols used in different research. In our work, at the time of sample collection, patients of successful isolation had no DENV-specific antibodies developed except for two patients with moderate titer (1:10) IgM antibodies. Our findings are that clinical samples collected between 0 and 6 days post symptom onset and before the development of neutralizing antibodies coincide with findings of other studies [16,45]. Other factors include the conditions of storage and transportation of specimens before arriving at the laboratory, where they were stored at 2 to 8 °C for up to 72 h and at −80 °C thereafter until isolation. We assume that samples coming from outpatient centers had longer storage and transportation time than specimens arriving from hospitals. We have no information on whether any temperature deviations happened during cold chain logistics. The number of freeze–thaw cycles of all specimens was limited to one before virus isolation.

## 5. Conclusions

Due to the rising temperatures and changes in climate conditions, the introduction of tropical arbovirus infections to new geographical areas could be expected, resulting in growing annual case numbers of imported infections [50]. Virus isolation from clinical specimens and the characterization of isolated DENV variants by whole-genome sequencing and phylogenetic analysis help the identification of the circulating dominant DENV genotypes and their connection with disease severity, which can further result in autochthonous transmission, even in Europe. The efficacy of virus isolation is influenced by multiple factors, according to our study viral load: time of sample collection, patient antibody status, specimen type, sample storage, and transportation conditions seem to strongly influence the outcomes of the procedure. Further investigation is needed to determine the optimal method and suitable sample materials. Virus isolation is important as isolated strains allow full viral genome sequencing and phylogenetic analysis that help to investigate the molecular epidemiology of outbreaks. In addition, they can be used in studies to help better understand virus pathogenicity or to test novel antiviral drugs or vaccine candidates [51]. Determining the specimen types that contain infective virus particles is important for identifying possible new risks of transmission. Throughout recent years, many autochthonous DENV epidemics have been recorded in Europe [52,53,54,55] that originated from an imported index case. Therefore, effective, and widespread vector-monitoring programs are essential in arbovirus surveillance. In addition to testing symptomatic patients, the screening of asymptomatic travelers who return from areas with DENV circulation should also be considered as the majority of DENV infections remain subclinical [2,3,4].

## Figures and Tables

**Table 1:** Patient symptoms, travel history, time of sample collection (days after symptom onset), serological results (detected: +, not detected: -), Ct values of samples, and the corresponding isolates.

Sample ID	Specimen Type	Time of Sampling *	Travel History	Symptoms	NS1	IgM	IgG	Ct Sample	Ct Isolate
102/2017	serum	3	Malaysia, Cambodia, Philippines	fever, rash, myalgia, leucopenia, arthralgia	+	-	-	25.88	22.97
686/2017	serum	2	Thailand	fever, nausea, myalgia, thrombocytopenia, leucopenia	+	-	-	21.34	19.08
5739/2017	serum	1	Borneo	fever	+	-	-	21.61	18.57
7030/2018	whole-blood	1	Malaysia	fever, myalgia	+	-	-	23.92	20.67
1266/2019	whole-blood	4	Thailand	fever, myalgia, rash	+	+	-	21.93	16.77
1354/2019	serum	3	Maldives	fever, rash, petechia	+	-	-	23.54	17.35
2202/2019	serum	2	Cambodia, Bali, Kuala Lumpur	fever	+	-	-	27.35	16.03
6484/2019	serum	1	Maldives	fever, arthralgia	+	-	-	24.04	20.84
34/2021	serum	0	Saint-Martin	fever, rash, petechia, mucosal bleeding, conjunctivitis	+	-	-	21.45	16.41
48/2021	serum	1	Saint-Martin	fever, collapse	+	-	-	17.60	15.96
9/2022	serum	6	Maldives	fever, rash, thrombocytopenia	+	+	-	24.81	15.90

* Day of sample collection after symptom onset. NS1, IgM, IgG: detected: +, not detected: -.

**Table 2:** Results of whole-genome sequencing of DENV isolates.

Sample ID	GenBank Accession Number	Geographical Region	Genotype Assignment	Reference Genomes Used for Mapping
102/2017	OP895913	Malaysia, Cambodia, Philippines	DENV-1 Genotype IV	LC128301.1
686/2017	OP895914	Thailand	DENV-1 Genotype I	OP410988.1
5739/2017	OP895916	Borneo	DENV-1 Genotype I	OP410988.1
7030/2018	OP895919	Malaysia	DENV-2 Genotype II— Cosmopolitan	NC_001474.2
1266/2019	OP895915	Thailand	DENV-1 Genotype I	OP410988.1
1354/2019	OP895928	Maldives	DENV-3 Genotype III	NC_001475.2
2202/2019	OP895918	Cambodia, Bali, Kuala Lumpur	DENV-2 Genotype II— Cosmopolitan	NC_001474.2
6484/2019	OP895929	Maldives	DENV-3 Genotype III	NC_001475.2
34/2021	OP895911	Saint Martin	DENV-1 Genotype V	EU482591.1
48/2021	OP895912	Saint Martin	DENV-1 Genotype V	EU482591.1
9/2022	OP895917	Maldives	DENV-2 Genotype II— Cosmopolitan	NC_001474.2

## Data Availability

Data is contained within the article or Supplementary Material.

## Supplementary Materials

The following supporting information can be downloaded at: https://www.mdpi.com/article/10.3390/diagnostics13050873/s1, Table S1: Detailed results of whole-genome sequencing of DENV isolates.

## References

[1] Roy S.K., Bhattacharjee S. (2021). Dengue virus: Epidemiology, biology, and disease aetiology. Can. J. Microbiol..

[2] Bhatt S., Gething P.W., Brady O.J., Messina J.P., Farlow A.W., Moyes C.L., Drake J.M., Brownstein J.S., Hoen A.G., Sankoh O. (2013). The global distribution and burden of dengue. Nature.

[3] Martina B.E., Koraka P., Osterhaus A.D. (2009). Dengue virus pathogenesis: An integrated view. Clin. Microbiol. Rev..

[4] Chen R.F., Yang K.D., Wang L., Liu J.W., Chiu C.C., Cheng J.T. (2007). Different clinical and laboratory manifestations between dengue haemorrhagic fever and dengue fever with bleeding tendency. Trans. R. Soc. Trop. Med. Hyg..

[5] Narayan R., Tripathi S. (2020). Intrinsic ADE: The Dark Side of Antibody Dependent Enhancement During Dengue Infection. Front. Cell. Infect. Microbiol..

[6] Shukla R., Ramasamy V., Shanmugam R.K., Ahuja R., Khanna N. (2020). Antibody-Dependent Enhancement: A Challenge for Developing a Safe Dengue Vaccine. Front. Cell. Infect. Microbiol..

[7] Dejnirattisai W., Jumnainsong A., Onsirisakul N., Fitton P., Vasanawathana S., Limpitikul W., Puttikhunt C., Edwards C., Duangchinda T., Supasa S. (2010). Cross-reacting antibodies enhance dengue virus infection in humans. Science.

[8] Lambrechts L., Scott T.W., Gubler D.J. (2010). Consequences of the expanding global distribution of *Aedes albopictus* for dengue virus transmission. PLoS Negl. Trop. Dis..

[9] European Centre for Disease Prevention and Control and European Food Safety Authority (2022). Mosquito Maps. Stockholm: ECDC. https://ecdc.europa.eu/en/disease-vectors/surveillance-and-disease-data/mosquito-maps.

[10] Liu-Helmersson J., Quam M., Wilder-Smith A., Stenlund H., Ebi K., Massad E., Rocklöv J. (2016). Climate Change and *Aedes* Vectors: 21st Century Projections for Dengue Transmission in Europe. EBioMedicine.

[11] European Medicines Agency Dengvaxia. https://www.ema.europa.eu/en/medicines/human/EPAR/dengvaxia.

[12] Thomas S.J., Yoon I.K. (2019). A review of Dengvaxia®: Development to deployment. Hum. Vaccin. Immunother..

[13] Muller D.A., Depelsenaire A.C., Young P.R. (2017). Clinical and Laboratory Diagnosis of Dengue Virus Infection. J. Infect. Dis..

[14] Klungthong C., Gibbons R.V., Thaisomboonsuk B., Nisalak A., Kalayanarooj S., Thirawuth V., Nutkumhang N., Mammen M.P., Jarman R.G. (2007). Dengue virus detection using whole blood for reverse transcriptase PCR and virus isolation. J. Clin. Microbiol..

[15] Humaidi M., Tien W.P., Yap G., Chua C.R., Ng L.C. (2021). Non-Invasive Dengue Diagnostics-The Use of Saliva and Urine for Different Stages of the Illness. Diagnostics.

[16] Vaughn D.W., Green S., Kalayanarooj S., Innis B.L., Nimmannitya S., Suntayakorn S., Rothman A.L., Ennis F.A., Nisalak A. (1997). Dengue in the early febrile phase: Viremia and antibody responses. J. Infect. Dis..

[17] Rathore A.P.S., St John A.L. (2020). Cross-Reactive Immunity Among Flaviviruses. Front. Immunol..

[18] Nagy A., Mezei E., Nagy O., Bakonyi T., Csonka N., Kaposi M., Koroknai A., Szomor K., Rigó Z., Molnár Z. (2019). Extraordinary increase in West Nile virus cases and first confirmed human Usutu virus infection in Hungary, 2018. Eurosurveillance.

[19] Nagy A., Nagy O., Tarcsai K., Farkas Á., Takács M. (2018). First detection of tick-borne encephalitis virus RNA in clinical specimens of acutely ill patients in Hungary. Ticks Tick Borne Dis..

[20] Santiago G.A., Vergne E., Quiles Y., Cosme J., Vazquez J., Medina J.F., Medina F., Colón C., Margolis H., Muñoz-Jordán J.L. (2013). Analytical and clinical performance of the CDC real time RT-PCR assay for detection and typing of dengue virus. PLoS Negl. Trop. Dis..

[21] ECDC EU Case Definitions. https://www.ecdc.europa.eu/en/all-topics/eu-case-definitions.

[22] Vilsker M., Moosa Y., Nooij S., Fonseca V., Ghysens Y., Dumon K., Pauwels R., Alcantara L.C., Vanden Eynden E., Vandamme A.M. (2019). Genome Detective: An automated system for virus identification from high-throughput sequencing data. Bioinformatics.

[23] Dengue Virus Typing Tool. https://www.genomedetective.com/app/typingtool/dengue/.

[24] GenBank Overview. https://www.ncbi.nlm.nih.gov/genbank/.

[25] BLAST: Basic Local Alignment Tool. https://blast.ncbi.nlm.nih.gov/Blast.cgi.

[26] MUSCLE: Multiple Sequence Alignment. https://www.ebi.ac.uk/Tools/msa/muscle/.

[27] Nicholas K.B., Nicholas H.B., Deerfield D.W. (1997). GeneDoc: Analysis and visualization of genetic variation. EMBNEW News.

[28] Tamura K., Stecher G., Kumar S. (2021). MEGA11: Molecular Evolutionary Genetics Analysis Version 11. Mol. Biol. Evol..

[29] OhAinle M., Balmaseda A., Macalalad A.R., Tellez Y., Zody M.C., Saborío S., Nuñez A., Lennon N.J., Birren B.W., Gordon A. (2011). Dynamics of dengue disease severity determined by the interplay between viral genetics and serotype-specific immunity. Sci. Transl. Med..

[30] Balmaseda A., Hammond S.N., Pérez L., Tellez Y., Saborío S.I., Mercado J.C., Cuadra R., Ro-cha J., Pérez M.A., Silva S. (2006). Serotype-specific differences in clinical manifestations of dengue. Am. J. Trop. Med. Hyg..

[31] Borges M.B., Marchevsky R.S., Mendes Y.S., Mendes L.G., Duarte A.C., Cruz M., de Filippis A.M.B., Vasconcelos P.F.C., Freire M., Homma A. (2018). Characterization of recent and minimally passaged Brazilian dengue viruses inducing robust infection in rhesus macaques. PLoS ONE.

[32] Fontaine A., Lequime S., Moltini-Conclois I., Jiolle D., Leparc-Goffart I., Reiner R.C., Lambrechts L. (2018). Epidemiological significance of dengue virus genetic variation in mosquito infection dynamics. PLoS Pathog..

[33] Leitmeyer K.C., Vaughn D.W., Watts D.M., Salas R., Villalobos I., Chacon D., Ramos C., Rico-Hesse R. (1999). Dengue virus structural differences that correlate with pathogenesis. J. Virol..

[34] Vaughn D.W., Green S., Kalayanarooj S., Innis B.L., Nimmannitya S., Suntayakorn S., Endy T.P., Raengsakulrach B., Rothman A.L., Ennis F.A. (2000). Dengue viremia titer, antibody response pattern, and virus serotype correlate with disease severity. J. Infect. Dis..

[35] Watts D.M., Porter K.R., Putvatana P., Vasquez B., Calampa C., Hayes C.G., Halstead S.B. (1999). Failure of secondary infection with American genotype dengue 2 to cause dengue haemorrhagic fever. Lancet.

[36] Shrestha D.B., Budhathoki P., Gurung B., Subedi S., Aryal S., Basukala A., Aryal B., Adhikari A., Poudel A., Yadav G.K. (2022). Epidemiology of dengue in SAARC territory: A systematic review and meta-analysis. Parasites Vectors.

[37] Hamel R., Surasombatpattana P., Wichit S., Dauvé A., Donato C., Pompon J., Vijaykrishna D., Liegeois F., Vargas R.M., Luplertlop N. (2019). Phylogenetic analysis revealed the co-circulation of four dengue virus serotypes in Southern Thailand. PLoS ONE.

[38] Rivera A., Adams L.E., Sharp T.M., Lehman J.A., Waterman S.H., Paz-Bailey G. (2020). Trav-el-Associated and Locally Acquired Dengue Cases-United States, 2010–2017. MMWR Morb. Mortal Wkly. Rep..

[39] Pagani G., Zanchetta N., Galimberti L., Oreni L., Passerini S., Giacomelli A., Cordier L., Gismondo M.R., Rizzardini G., Galli M. (2020). Imported dengue fever: A 16-years retrospective analysis in Milan (Italy) and a brief review of the European literature. Infez. Med..

[40] Riccò M., Peruzzi S., Balzarini F., Zaniboni A., Ranzieri S. (2022). Dengue Fever in Italy: The "Eternal Return" of an Emerging Arboviral Disease. Trop. Med. Infect. Dis..

[41] Suppiah J., Ching S.M., Amin-Nordin S., Mat-Nor L.A., Ahmad-Najimudin N.A., Low G.K., Abdul-Wahid M.Z., Thayan R., Chee H.Y. (2018). Clinical manifestations of dengue in relation to dengue serotype and genotype in Malaysia: A retrospective observational study. PLoS Negl. Trop. Dis..

[42] Patil J.A., Cherian S., Walimbe A.M., Bhagat A., Vallentyne J., Kakade M., Shah P.S., Cecilia D. (2012). Influence of evolutionary events on the Indian subcontinent on the phylogeography of dengue type 3 and 4 viruses. Infect. Genet Evol..

[43] Cecilia D., Patil J.A., Kakade M.B., Walimbe A., Alagarasu K., Anukumar B., Abraham A. (2017). Emergence of the Asian genotype of DENV-1 in South India. Virology.

[44] Malik H.A.M., Abid F., Wahiddin M.R., Waqas A. (2021). Modeling of internal and external factors affecting a complex dengue network. Chaos Solitons Fractals.

[45] Kar M., Nisheetha A., Kumar A., Jagtap S., Shinde J., Singla M.M.S., Pandit A., Chandele A., Kabra S.K., Krishna S. (2019). Isolation and molecular characterization of dengue virus clinical isolates from pediatric patients in New Delhi. Int. J. Infect. Dis..

[46] Scott R.M., Nisalak A., Cheamudon U., Seridhoranakul S., Nimmannitya S. (1980). Isolation of dengue viruses from peripheral blood leukocytes of patients with hemorrhagic fever. J. Infect. Dis..

[47] Wang H.L., Lin K.H., Yueh Y.Y., Chow L., Wu Y.C., Chen H.Y., Sheu M.M., Chen W.J. (2000). Efficient diagnosis of dengue infections using patients’ peripheral blood leukocytes and serum/plasma. Intervirology.

[48] De Paula S.O., Lopes da Fonseca B.A. (2002). Optimizing dengue diagnosis by RT-PCR in IgM-positive samples: Comparison of whole blood, buffy-coat and serum as clinical samples. J. Virol. Methods.

[49] Waterman S.H., Kuno G., Gubler D.J., Sather G.E. (1985). Low rates of antigen detection and virus isolation from the peripheral blood leukocytes of dengue fever patients. Am. J. Trop. Med. Hyg..

[50] Weaver S.C., Reisen W.K. (2010). Present and future arboviral threats. Antivir. Res..

[51] Pinheiro-Michelsen J.R., Souza R.D.S.O., Santana I.V.R., da Silva P.S., Mendez E.C., Luiz W.B., Amorim J.H. (2020). Anti-dengue Vaccines: From Development to Clinical Trials. Front. Immunol..

[52] Lazzarini L., Barzon L., Foglia F., Manfrin V., Pacenti M., Pavan G., Rassu M., Capelli G., Montarsi F., Martini S. (2020). First autochthonous dengue outbreak in Italy, August 2020. Eurosurveillance.

[53] La Ruche G., Souarès Y., Armengaud A., Peloux-Petiot F., Delaunay P., Desprès P., Lenglet A., Jourdain F., Leparc-Goffart I., Charlet F. (2010). First two autochthonous dengue virus infections in metropolitan France, September 2010. Eurosurveillance.

[54] Alves M.J., Fernandes P.L., Amaro F., Osório H., Luz T., Parreira P., Andrade G., Zé-Zé L., Zeller H. (2013). Clinical presentation and laboratory findings for the first autochthonous cases of dengue fever in Madeira island, Portugal, October 2012. Eurosurveillance.

[55] European Centre for Disease Prevention and Control (ECDC) Autochthonous Cases of Dengue in Spain and France 1 October 2019. https://www.ecdc.europa.eu/sites/portal/files/documents/RRA-dengue-in-Spain-and-France.pdf.

